# The H29D Mutation Does Not Enhance Cytosolic Ca^2+^ Activation of the Cardiac Ryanodine Receptor

**DOI:** 10.1371/journal.pone.0139058

**Published:** 2015-09-25

**Authors:** Zhichao Xiao, Wenting Guo, Siobhan M. Wong King Yuen, Ruiwu Wang, Lin Zhang, Filip Van Petegem, S. R. Wayne Chen

**Affiliations:** 1 Libin Cardiovascular Institute of Alberta, Department of Physiology & Pharmacology, University of Calgary, Calgary, Alberta, Canada T2N 4N1; 2 Libin Cardiovascular Institute of Alberta, Department of Biochemistry & Molecular Biology, University of Calgary, Calgary, Alberta, Canada T2N 4N1; 3 Department of Biochemistry and Molecular Biology, University of British Columbia, 2350 Health Sciences Mall, Vancouver, Canada V6T 1Z3; University of Canberra, AUSTRALIA

## Abstract

The N-terminal domain of the cardiac ryanodine receptor (RyR2) harbors a large number of naturally occurring mutations that are associated with stress-induced ventricular tachyarrhythmia and sudden death. Nearly all these disease-associated N-terminal mutations are located at domain interfaces or buried within domains. Mutations at these locations would alter domain-domain interactions or the stability/folding of domains. Recently, a novel RyR2 mutation H29D associated with ventricular arrhythmia at rest was found to enhance the activation of single RyR2 channels by diastolic levels of cytosolic Ca^2+^. Unlike other N-terminal disease-associated mutations, the H29D mutation is located on the surface of the N-terminal domain. It is unclear how this surface-exposed H29D mutation that does not appear to interact with other parts of the RyR2 structure could alter the intrinsic properties of the channel. Here we carried out detailed functional characterization of the RyR2-H29D mutant at the molecular and cellular levels. We found that the H29D mutation has no effect on the basal level or the Ca^2+^ dependent activation of [^3^H]ryanodine binding to RyR2, the cytosolic Ca^2+^ activation of single RyR2 channels, or the cytosolic Ca^2+^- or caffeine-induced Ca^2+^ release in HEK293 cells. In addition, the H29D mutation does not alter the propensity for spontaneous Ca^2+^ release or the thresholds for Ca^2+^ release activation or termination. Furthermore, the H29D mutation does not have significant impact on the thermal stability of the N-terminal region (residues 1–547) of RyR2. Collectively, our data show that the H29D mutation exerts little or no effect on the function of RyR2 or on the folding stability of the N-terminal region. Thus, our results provide no evidence that the H29D mutation enhances the cytosolic Ca^2+^ activation of RyR2.

## Introduction

The cardiac ryanodine receptor (RyR2) is an essential component of excitation-contraction coupling in the heart [1,2]. RyR2 is also a critical player in the pathogenesis of cardiac arrhythmias and cardiomyopathies. [2–5]. To date, more than 150 naturally occurring, disease-associated mutations in RyR2 have been identified. However, the functional consequences of most of these disease-associated RyR2 mutations are completely unknown. Only a small fraction of these RyR2 mutations have been functionally characterized. To improve the diagnosis and treatment of RyR2-linked diseases, it is imperative to understand the functional impact of disease-associated RyR2 mutations.

Recent major advances in defining the three-dimensional (3D) structure of RyR have provided invaluable and detailed insights into the molecular mechanisms of disease-associated RyR mutations. For instance, high-resolution structures of the N-terminal region of RyR have recently been solved [6–16]. The N-terminal region of RyR2 consists of three domains: two domains that form β-trefoils (domain A residues 1–217), domain B (residues 218–409), and domain C (residues 410–543) that is part of a longer α-solenoid in intact RyRs [14–16]. Mapping of more than 50 disease-associated mutations into the 3D structure of the N-terminal domains has revealed that all of the disease-associated mutations in this region are located at domain interfaces or buried within individual domains [8,12,13]. In RyR2, these domain interfaces appear particularly important: a central chloride ion is coordinated by residues of all three domains, and disruption of the binding through disease-causing mutations results in domain reorientations [8,12,13]. These observations have led to the proposition that disease-causing mutations in the N-terminal region destabilize domain interfaces or disrupt the folding of individual domains, which would impair domain-domain interactions and structural changes that are required for proper gating of the channel [6,8,9,11–13,17]. In line with this hypothesis, we have shown that a number of disease-associated mutations in the N-terminal region alter the activation and/or inactivation of the RyR2 channel [18–22].

A novel RyR2 mutation, H29D, has recently been identified and is believed to be associated with short-coupled polymorphic ventricular tachycardia at rest [23]. Functional characterization using single channel recordings in planar lipid bilayers revealed that single H29D mutant channels exhibited enhanced cytosolic Ca^2+^ activation at diastolic cytosolic Ca^2+^ concentrations compared to single RyR2 WT channels [23]. It was proposed that the H29D mutation causes a 'leaky' channel at low cytosolic Ca^2+^ concentrations, and that 'leaky' RyR2 may be a mechanism for polymorphic ventricular tachycardia at rest [23]. However, unlike all other known N-terminal disease mutations [8,12,13], the H29D mutation is not located at a domain interface or within a domain. The H29D mutation is located in a loop between beta-sheet 1 and 2, and mapped to the surface of the RyR2 structure that is facing the transverse tubular membrane [14–16]. Moreover, the H29 side chain has been shown to display great flexibility in multiple crystal structures of the RyR2 N-terminal region [12], further indicating that it forms no significant interactions with any other residues. How then could the H29D mutation affect intrinsic properties of the RyR2 channel? To address this question, in the present study, we characterized the H29D mutant at the molecular and cellular levels using a number of functional and biochemical assays. We found that the H29D mutation did not alter the basal level or the Ca^2+^ dependence of [^3^H]ryanodine binding to RyR2. Single channel analysis showed that the H29D mutation did not affect cytosolic Ca^2+^ activation of single RyR2 channels. The H29D mutation also had no effect on the activation of RyR2 by caffeine or the propensity for store-overload induced Ca^2+^ release (SOICR). The H29D mutation also did not alter the cytosolic Ca^2+^ dependence of Ca^2+^ release or the activation or termination threshold for SOICR. Furthermore, the H29D mutation had no effect on the expression of the RyR2 channel or the thermal stability of the N-terminal domains of RyR2. Collectively, our data indicate that the H29D mutation does not significantly alter the intrinsic properties of RyR2. Our results do not support the notion that the H29D mutation causes a 'leaky' RyR2 channel.

## Materials and Methods

### Construction of the RyR2-H29D mutation

Point mutation, H29D, in the mouse RyR2 was generated by the overlap extension method using PCR [24,25]. Briefly, a NheI-AflII fragment with mutation H29D was produced by overlapping PCR. The NheI-AflII fragment was removed from the PCR product and used to replace the corresponding wild-type (WT) fragment in the full-length RyR2 cDNA in the expression plasmid pcDNA5. The mutation and sequence of the PCR product were confirmed by DNA sequencing.

### Generation of stable, inducible HEK293 cell lines

Stable, inducible HEK293 cell lines expressing RyR2-WT or the RyR2-H29D mutant were generated using the Flp-In T-REx Core Kit from Invitrogen [26]. Briefly, Flp-In T-REx-293 cells were co-transfected with the inducible expression vector pcDNA5/FRT/TO containing the WT or mutant RyR2 cDNAs and the pOG44 vector encoding the Flp recombinase in 1: 5 ratios using the Ca^2+^ phosphate precipitation method. The transfected cells were washed with PBS 24 h after transfection followed by a change into fresh media for 24 h. The cells were then washed again with PBS, harvested, and plated onto new dishes. After the cells had attached (~4 h), the growth medium was replaced with a selection medium containing 200 μg/ml hygromycin (Invitrogen). The selection medium was changed every 3–4 days until the desired number of cells was grown. The hygromycin-resistant cells were pooled, aliquoted, and stored at -80°C. These positive cells are believed to be isogenic, because the integration of RyR2 cDNA is mediated by the Flp recombinase at a single FRT site. The handling and storage of the human embryonic kidney 293 (HEK293) cell line were approved by the Institutional Biosafety Committee at the University of Calgary.

### [^3^H]ryanodine binding

HEK293 cells were plated in 100 mm tissue culture dishes at ~10% confluence 18~20 h before transfection with RyR2 WT or H29D mutant cDNA. After transfection for 24 h, the cells were harvested and lysed in lysis buffer containing 25 mM Tris, 50 mM HEPES, pH 7.4, 137 mM NaCl, 1% CHAPS, 0.5% egg phosphatidylcholine, 2.5 mM DTT, and a protease inhibitor mix (1 mM benzamidine, 2 μg/ml leupeptin, 2 μg/ml pepstatin A, 2 μg/ml aprotinin, and 0.5 mM PMSF) on ice for 60 min. Cell lysate was obtained after removing the unsolubilized materials by centrifugation twice in a microcentrifuge at 4°C for 30 min each. Equilibrium [^3^H]ryanodine binding to cell lysates was performed as described previously [27] with some modifications. [^3^H]Ryanodine binding was carried out in a total volume of 300 μl binding solution containing 30 μl of cell lysate, 100 mM KCl, 25 mM Tris, 50 mM Hepes (pH 7.4), 5 nM [^3^H]ryanodine and CaCl_2_ to set free [Ca^2+^] from pCa 9.89 to pCa 4 and a protease inhibitor mix at 37°C for 20 min. Ca^2+^/EGTA ratio was calculated using the computer program of Fabiato and Fabiato [28]. The binding mix was diluted with 5 ml of ice-cold washing buffer containing 25 mM Tris, pH 8.0, and 250mM KCl and immediately filtered through Whatman GF/B filters presoaked with 1% polyethylenimine. The filters were washed three times, and the radioactivity associated with the filters was determined by liquid scintillation counting. Nonspecific binding was determined by measuring [^3^H]ryanodine binding in the presence of 50 μM unlabeled ryanodine. All binding assays were done in duplicate.

### Western blotting

HEK293 cell lines grown for 24 h after induction were washed with PBS (137 mM NaCl, 8 mM Na_2_HPO4, 1.5 mM KH_2_PO_4_, 2.7 mM KCl) plus 2.5 mM EDTA and harvested in the same solution by centrifugation for 8 min at 700 x g in an IEC Centra-CL2 centrifuge. The cells were then washed with PBS without EDTA and centrifuged again at 700 x g for 8 min. The PBS washed cells were solubilized in a lysis buffer containing 25 mM Tris, 50 mM Hepes (pH 7.4), 137 mM NaCl, 1% CHAPS, 0.5% soybean phosphatidylcholine, 2.5 mM DTT, and a protease inhibitor mix (1 mM benzamidine, 2 μg/ml leupeptin, 2 μg/ml pepstatin A, 2 μg/ml aprotinin, 0.5 mM PMSF). This mixture was incubated on ice for 1 hr. Cell lysate was obtained by centrifuging twice at 16,000 x g in a microcentrifuge at 4°C for 30 min to remove unsolubilized materials. The RyR2 WT and H29D mutant proteins were subjected to 6% SDS-PAGE [29] and transferred to nitrocellulose membranes at 45 V for 18–20 h at 4°C in the presence of 0.01% SDS [30]. The nitrocellulose membranes containing the transferred proteins were blocked for 30 min with PBS containing 0.5% Tween-20 and 5% skimmed-milk powder. The blocked membrane was incubated with the anti-RyR antibody (34c) (1:1000) and then incubated with the secondary anti-mouse IgG (H&L) antibodies conjugated to horseradish peroxidase (1:20000). After washing for 5 min, three times, the bound antibodies were detected using an enhanced chemiluminescence kit from Pierce.

### Purification of recombinant RyR2 proteins for single channel analysis

Recombinant RyR2 WT and H29D mutant channels were purified from cell lysate from HEK293 cells transfected with the RyR2 WT or the H29D mutant cDNA by sucrose density gradient centrifugation as described previously [18,27]. Briefly, RyR2 WT or the H29D mutant cell lysates (2.5 ml) were layered on top of a 5 ml (7.5–25%, wt/wt) linear sucrose gradient containing 25 mM Tris, 50 mM HEPES, pH 7.4, 0.3 M NaCl, 0.1 mM CaCl_2_, 0.3 mM EGTA, 0.25 mM PMSF, 4 μg/ml leupeptin, 5 mM DTT, 0.3% CHAPS, and 0.16% synthetic phosphatidylcholine. The gradient was centrifuged at 29,000 rpm in Beckman SW-41 rotor at 4°C for 17 h. Fractions of 0.7 ml each were collected. Peak fractions containing RyR proteins, as determined by immunoblotting, were pooled, aliquoted, and stored at −80°C.

### Single channel recordings in planar lipid bilayers

Single channel recordings were performed as described previously [26]. Briefly, heart phosphatidylethanolamine (50%) and brain phosphatidylserine (50%) (Avanti Polar Lipids), dissolved in chloroform, were combined and dried under nitrogen gas and resuspended in 30 **μ**l of *n*-decane at a concentration of 12 mg lipid per ml. Bilayers were formed across a 250 μm hole in a Delrin partition separating two chambers. The trans chamber (800 μl) was connected to the head stage input of an Axopatch 200A amplifier (Axon Instruments, Austin, TX). The cis chamber (1.2 ml) was held at virtual ground. A symmetrical solution containing 250 mM KCl and 25 mM Hepes (pH 7.4) was used for all recordings, unless indicated otherwise. A 4-μl aliquot (≈ 1 μg of protein) of the sucrose density gradient-purified recombinant RyR2 WT or the H29D mutant channels was added to the cis chamber. Single channels were incorporated into the bilayers in the recording solution without the addition of EGTA or Ca^2+^. Spontaneous channel activity was tested for sensitivity to EGTA and Ca^2+^ by adding EGTA (200 μM) to either the *cis* or *trans* chamber to determine the orientation of the incorporated channel. The chamber to which the addition of EGTA inhibited the activity of the incorporated channel presumably corresponds to the cytosolic side of the Ca^2+^ release channel. The EGTA-inhibited channel was then reactivated by the addition of various concentrations of Ca^2+^ (50–300 μM). The direction of single channel currents was always measured from the luminal to the cytosolic side of the channel, unless mentioned otherwise. Recordings were filtered at 2,500 Hz. Data analyses were carried out using the pclamp 8.1 software package (Axon Instruments). Free Ca^2+^ concentrations were calculated using the computer program of Fabiato and Fabiato [28].

### Caffeine-induced Ca^2+^ release in HEK293 cells

The free cytosolic Ca^2+^ concentration in transfected HEK293 cells was measured using the fluorescence Ca^2+^ indicator dye Fluo-3 (Molecular Probes) [26]. HEK293 cells grown on 100-mm tissue culture dishes for 18–20 h after subculture were transfected with 12–16 μg of RyR2 WT or the H29D mutant cDNA. Cells grown for 18–20 h after transfection were washed four times with PBS and incubated in KRH (Krebs–Ringer–Hepes. 125 mM NaCl, 5 mM KCl, 1.2 mM KH_2_PO_4_, 6 mM glucose, 1.2 mM MgCl_2_, 2 mM CaCl_2_ and 25 mM HEPES, pH 7.4) buffer without MgCl_2_ and CaCl_2_ at room temperature for 40 min and at 37°C for 40 min. After being detached from culture dishes by pipetting, cells were collected by centrifugation at 1,000 rpm for 2 min in a Beckman TH-4 rotor. Cell pellets were loaded with 10 μM Fluo-3 AM in high glucose Dulbecco's Modified Eagle Medium at room temperature for 60 min, followed by washing with KRH buffer plus 2mM CaCl_2_ and 1.2mM MgCl_2_ (KRH+ buffer) three times and resuspended in 150 μl KRH+ buffer plus 0.1 mg/ml BSA and 250 μM sulfinpyrazone. The Fluo-3 AM loaded cells were added to 2 ml (final volume) KRH+ buffer in a cuvette. The fluorescence intensity of Fluo-3 at 530 nm was measured before and after repeated additions or single additions of various concentrations of caffeine (0.025-5mM) in an SLM-Aminco series 2 luminescence spectrometer with 480nm excitation at 25°C (SLM Instruments). The peak levels of each caffeine-induced Ca^2+^ release were determined and normalized to the highest level (100%) of caffeine-induced Ca^2+^ release for each experiment.

### Single Cell Ca^2+^ imaging (cytosolic Ca^2+^ measurements) in HEK293 cells

Cytosolic Ca^2+^ levels in stable, inducible HEK293 cells expressing RyR2 WT or the H29D mutant were monitored using single-cell Ca^2+^ imaging and the fluorescent Ca^2+^ indicator dye Fura-2 as described previously [18,26,31]. Briefly, cells grown on glass coverslips for 8-18h after induction (as indicated) by 1 μg/ml tetracycline (Sigma) were loaded with 5 μM Fura-2, AM in KRH buffer (125 mM NaCl, 5 mM KCl, 6 mM glucose, 1.2 mM MgCl_2_ and 25 mM Hepes, pH 7.4) plus 0.02% pluronic F-127 and 0.1 mg/ml BSA for 20 min at room temperature (23°C). The coverslips were then mounted in a perfusion chamber (Warner Instruments) on an inverted microscope (Nikon TE2000-S). The cells were perfused continuously with KRH buffer containing increasing extracellular Ca^2+^ concentrations (0, 0.1, 0.2, 0.3, 0.5, 1.0 and 2.0 mM). Caffeine (10 mM) was applied at the end of each experiment to confirm the expression of active RyR2 channels. Time-lapse images (0.25 frame/s) were captured and analyzed with Compix Simple PCI 6 software. Fluorescence intensities were measured from regions of interest centered on individual cells. Only cells that responded to caffeine were analyzed. The filters used for Fura-2 imaging were λex = 340±26 nm and 387±11 nm, and λem = 510±84 nm with a dichroic mirror (410 nM).

### Single cell Ca^2+^ imaging (luminal Ca^2+^ measurements) in HEK293 cells

Luminal Ca^2+^ levels in HEK293 cells expressing RyR2 WT or mutants were measured using single-cell Ca^2+^imaging and the FRET (fluorescence resonance energy transfer)-based ER luminal Ca^2+^-sensitive cameleon protein D1ER as described previously[32,33]. The cells were grown to 95% confluence in a 75 cm^2^ flask, passaged with PBS and plated in 100-mm-diameter tissue culture dishes at ~10% confluence 18–20 h before transfection with D1ER cDNA using the calcium phosphate precipitation method. After transfection for 24 h, the growth medium was then changed to an induction medium containing 1 μg/ml tetracycline. In intact cell studies, after induction for ~22 h, the cells were perfused continuously with KRH buffer containing various concentrations of CaCl_2_ (0, 1 and 2 mM) and tetracaine (1 mM) for estimating the store capacity or caffeine (20 mM) for estimating the minimum store level by depleting the ER Ca^2+^ stores at room temperature (23°C). In permeabilized cell studies, the cells were first permeabilized by 50 μg/ml saponin [34] in the incomplete intracellular-like medium (ICM) at room temperature (23°C) (The incomplete ICM contains 125 mM KCl, 19 mM NaCl, and 10 mM HEPES, pH 7.4, with KOH) for 3–4 min. The cells were then switched to complete ICM (incomplete ICM plus 2 mM ATP, 2 mM MgCl_2_, and 0.05 mM EGTA, and 100nM free Ca^2+^, pH7.4, with KOH) for 5–6min to remove saponin. The permeabilized cells were then perfusesd with various concentrations of Ca^2+^ (0.1, 0.2, 0.4, 1 and 10 μM) followed by tetracaine (1 mM) for estimating the store capacity and caffeine (10 mM) for estimating the minimum store level by depleting the ER Ca^2+^ stores. Images were captured with Compix Simple PCI 6 software every 2 s using an inverted microscope (Nikon TE2000-S) equipped with an S-Fluor 20×/0.75 objective. The filters used for D1ER imaging were λex = 436±20 nm for CFP and λex = 500±20 nm for YFP, and λem = 465±30 nm for CFP and λem = 535±30 nm for YFP with a dichroic mirror (500 nm). The amount of FRET was determined from the ratio of the light emission at 535 and 465 nm.

### Purification of RyR2ABC mutant protein

Mouse RyR2 cDNA (encoding residues 1–547), was expressed in *Escherichia coli* Rosetta (DE3) pLacI cells (Novagen) using a modified pET28 vector as previously described [12,13]. The H29D mutation was introduced by the QuikChange protocol (Stratagene). Cells expressing the H29D mutant were lysed by sonication in buffer A (250mM KCl and 10mM HEPES buffer, pH7.4) supplemented with 20mM imidazole and 50μg/ml DNase I, 50μg/ml lysozyme, 7mM β-mercaptoethanol (bME), 1mM phenylmethylsulphonyl fluoride, and 10%(vol/vol) glycerol The lysate was applied to 2x5ml HisTrap columns (GE Healthcare), washed with 15 column volumes (CVs) of buffer A and eluted with 80% (vol/vol) buffer B (250mM KCl and 500mM imidazole, pH 7.4). The protein was dialyzed against buffer A plus 7mM bME and was cleaved simultaneously with recombinant TEV protease. The protein was then applied to a 25ml amylose column (New England Biolabs), washed with 10 CVs of buffer A and eluted with buffer C (buffer A plus 10mM maltose). The protein was applied to a 25ml Talon column (ClonTech) in buffer A and then run on a Q Sepharose High Performance column (GE Healthcare) using in buffer D (50mM KCl, 10mM Tris pH 8.0 and 7mM bME) and eluted with a gradient of 0% to 20% buffer E (1M KCl, 10mM Tris, pH 8.0 and 7mM bME). The protein was subsequently applied to a HiLoad 16/10 Phenyl Sepharose HP column (GE Healthcare) in buffer E and eluted with a gradient of 100% to 0% buffer D. Lastly, the protein was run on a HiLoad 16/60 Superdex 200 prep grade gel filtration column (GE Healthcare) in buffer consisting of 250mM KCl, 10mM HEPES (pH7.4), and 7mM bME. The protein was concentrated to 12mg/ml using Amicon concentrators (10kDa molecular mass cutoff; Millipore) and used fresh. The wild type RyR2 (1–547) protein was purified in a similar manner with a minor difference: the HiLoad Q-Sepharose HP column was omitted.

### Thermal melt analysis

The protein melting curves were measured using thermofluor experiments [35] as described before for other RyR constructs [7–9,12,13]. Briefly, samples for melting curves contained 50 μl of purified protein at 0.3mg/ml and 1x SYPRO Orange solution (Invitrogen) using manufacturer’s instructions in buffer consisting of 150mM KCl, 10mM HEPES (pH7.4), and 14mM bME. The melts were obtained in a DNA engine opticon 2 real-time PCR machine (BIORAD) using the SYBR green filter option. The temperature was changed from 25°C to 95°C in 0.5°C steps. At every step, the temperature was kept constant for 15s. The melting temperatures were obtained by averaging the peak points in the first derivatives of the melt curves from five replicate runs.

### Statistical analysis

All values shown are mean ± SEM unless indicated otherwise. To test for differences between groups, we used Student's *t* test (2-tailed). A *P* value <0.05 was considered to be statistically significant.

## Results

### The RyR2-H29D mutation has no effect on the basal level or the Ca^2+^ dependence of [^3^H]ryanodine binding

**Fig 1:**
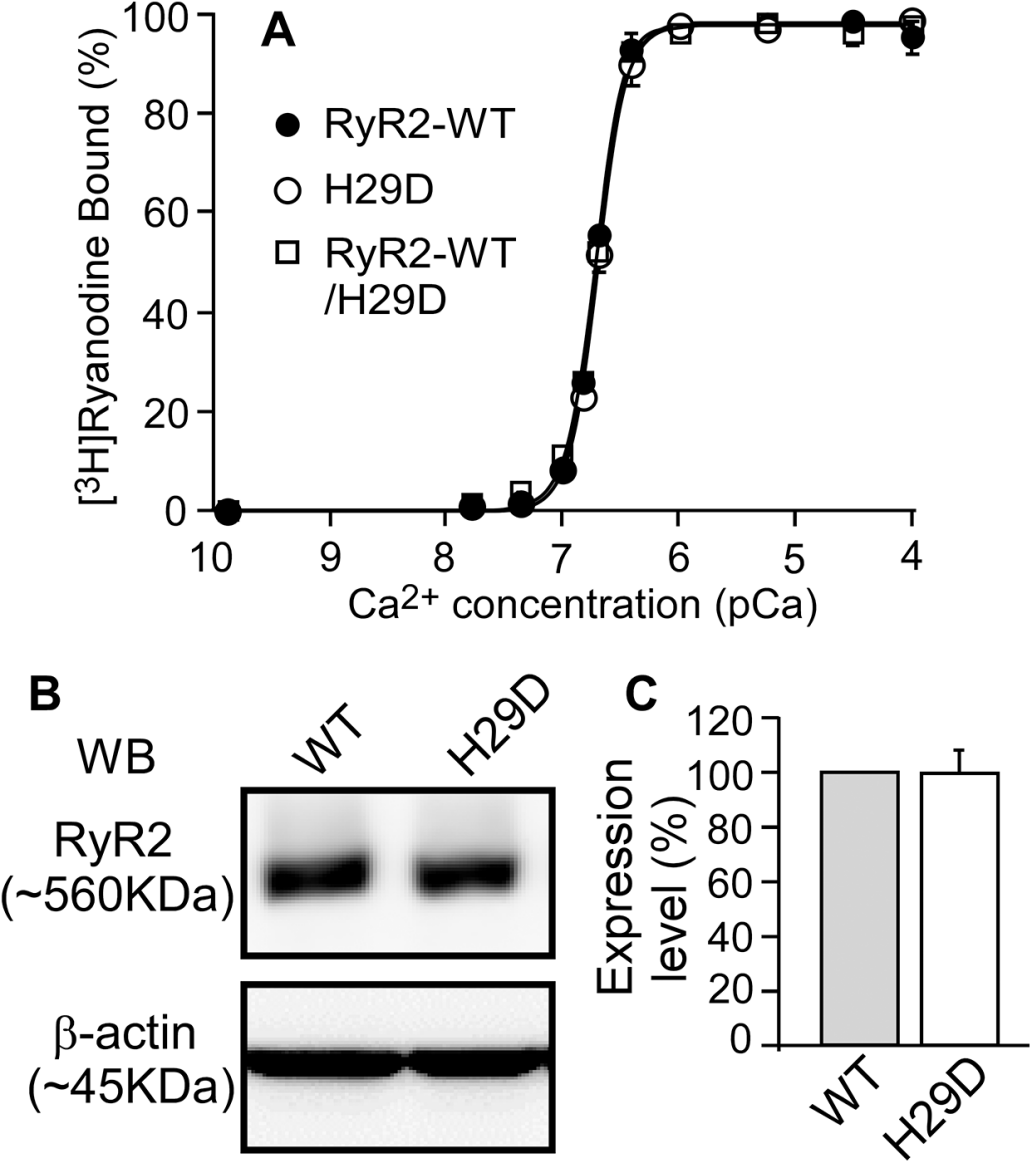
Effect of the H29D mutation on the Ca^2+^ dependent activation of [^3^H]ryanodine binding to RyR2. (A) [^3^H]ryanodine binding to cell lysate from HEK293 cells expressing the RyR2 WT or the H29D mutant was carried out at various Ca^2+^ concentrations (~0.2 nM to 0.1 mM), 100 mM KCl, and 5 nM [^3^H]ryanodine. Amounts of [^3^H]ryanodine binding at various Ca^2+^ concentrations were normalized to the maximal binding (100%). Data points shown are mean ± SEM from 3 separate experiments. (B,C) Immuno-blotting of RyR2 WT and the H29D mutant from the same amount of cell lysates using the anti-RyR antibody (34c) (n = 3).

Since ryanodine only binds to the open state of the RyR channel, [^3^H]ryanodine binding has commonly been used as an index for the open probability (Po) of RyR. Furthermore, because [^3^H]ryanodine, once it is bound to RyR, dissociates from the channel very slowly, equilibrium [^3^H]ryanodine binding assay performed over several hours is highly sensitive to detect RyR openings especially under conditions where the Po of the channel is very low. Therefore, we employed [^3^H]ryanodine binding assay to determine the impact of the H29D mutation on the basal activity of RyR2 at low Ca^2+^ concentrations and the Ca^2+^ dependent activation of the channel. As shown in Fig 1, the H29D mutation had no significant effect on [^3^H]ryanodine binding to RyR2 at low Ca^2+^ concentrations (45–150 nM) (Fig 1A). Furthermore, there was no significant difference in the EC_50_ of Ca^2+^ dependent activation of [^3^H]ryanodine binding between the RyR2 WT (0.193 ± 0.002 μM) and H29D mutant (0.203 ± 0.008 μM) (P = 0.338) (Fig 1A). We also performed [^3^H]ryanodine binding to lysate from HEK293 cells co-transfected with RyR2 WT and H29D (in 1:1 ratio) to mimic the heterozygous expression of H29D mutant in patients (i.e. H29D^+/-^). We found that H29D^+/-^ does not affect the EC_50_ of Ca^2+^ dependent activation of [^3^H]ryanodine binding (0.200 ± 0.002 μM) (P = 0.914). It should be noted that the expression level of the RyR2 WT and H29D mutant are similar (Fig 1B and 1C). Thus, our data indicate that the H29D mutation does not enhance the openings of the RyR2 channel at low Ca^2+^ concentrations or the sensitivity of the channel to Ca^2+^ activation.

### The H29D mutation does not alter the cytosolic Ca^2+^ activation of single RyR2 channels

**Fig 2:**
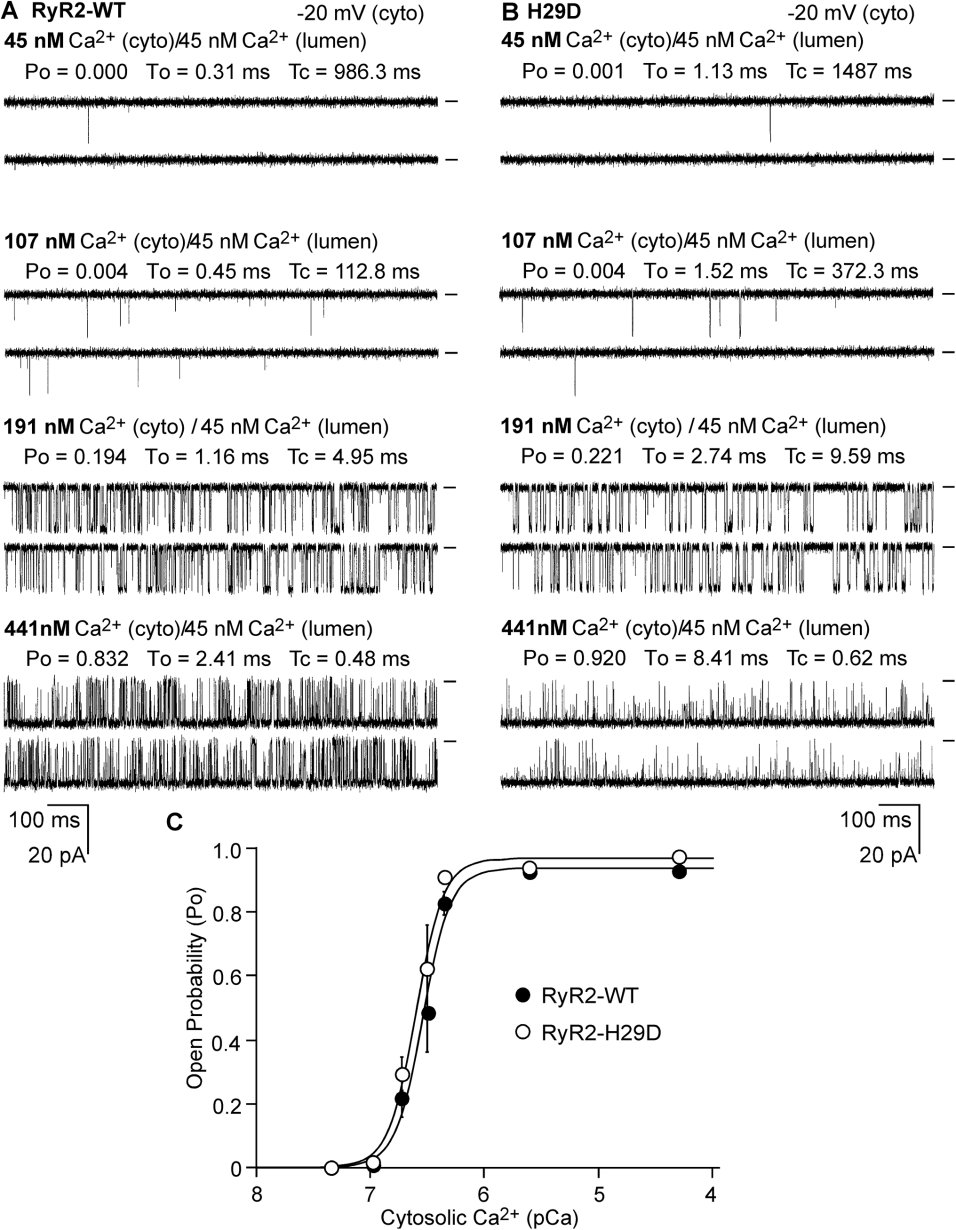
Effect of the H29D mutation on cytosolic Ca^2+^ activation of single RyR2 channels. Single channel activities of RyR2 WT (A) and H29D (B) were recorded in a symmetrical recording solution containing 250 mM KCl and 25 mM Hepes (pH 7.4). EGTA was added to either the *cis* or *trans* chamber to determine the orientation of the incorporated channel. The side of the channel to which an addition of EGTA inhibited the activity of the incorporated channel presumably corresponds to the cytoplasmic face. The Ca^2+^ concentration on both the cytoplasmic and the luminal face of the channel was adjusted to ~45 nM. The cytosolic Ca^2+^ concentration was then increased to various levels by an addition of aliquots of CaCl_2_ solution. Recording potential was -20mV. Openings are downward, and baselines are indicated (short bars). Open probability (Po), mean open time (To), and mean closed time (Tc) are shown. The relationships between Po and cytosolic Ca^2+^ concentrations (pCa) of single RyR2 WT (filled circles) and H29D mutant (open circles) channels are shown in panel C. Data points shown are mean ± SEM from 7 RyR2 WT and 5 H29D single channels.

To directly assess the impact of the H29D mutation on the cytosolic Ca^2+^ activation of RyR2, we determined the response of single RyR2 WT and the H29D mutant channels to various cytosolic Ca^2+^ concentrations (45nM to 50 μM) using single channel recordings in planar lipid bilayers. As shown in Fig 2, both single RyR2 WT and H29D mutant channels displayed similar open probability (Po) at low cytosolic Ca^2+^ levels (Fig 2A and 2B). There were no significant differences in Po between single RyR2 WT and H29D mutant channels at each cytosolic Ca^2+^ concentration tested (Fig 2C). Furthermore, there were also no significant differences in the gating behavior between single RyR2 WT and H29D mutant channels. The mean open times were 2.67 ± 0.65 ms for WT and 3.87 ± 0.39 ms for H29D (*P* = 0.148), and the mean closed time were 17.91 ± 7.20 ms for WT and 11.06 ± 3.20 for H29D (*P* = 0.409). Thus, the H29D mutation does not significantly affect the Po or the gating of the RyR2 channel at low cytosolic Ca^2+^ concentrations or the response of the channel to cytosolic Ca^2+^ activation.

### The H29D mutation does not affect cytosolic Ca^2+^ regulation of Ca^2+^ release in HEK293 cells

**Fig 3:**
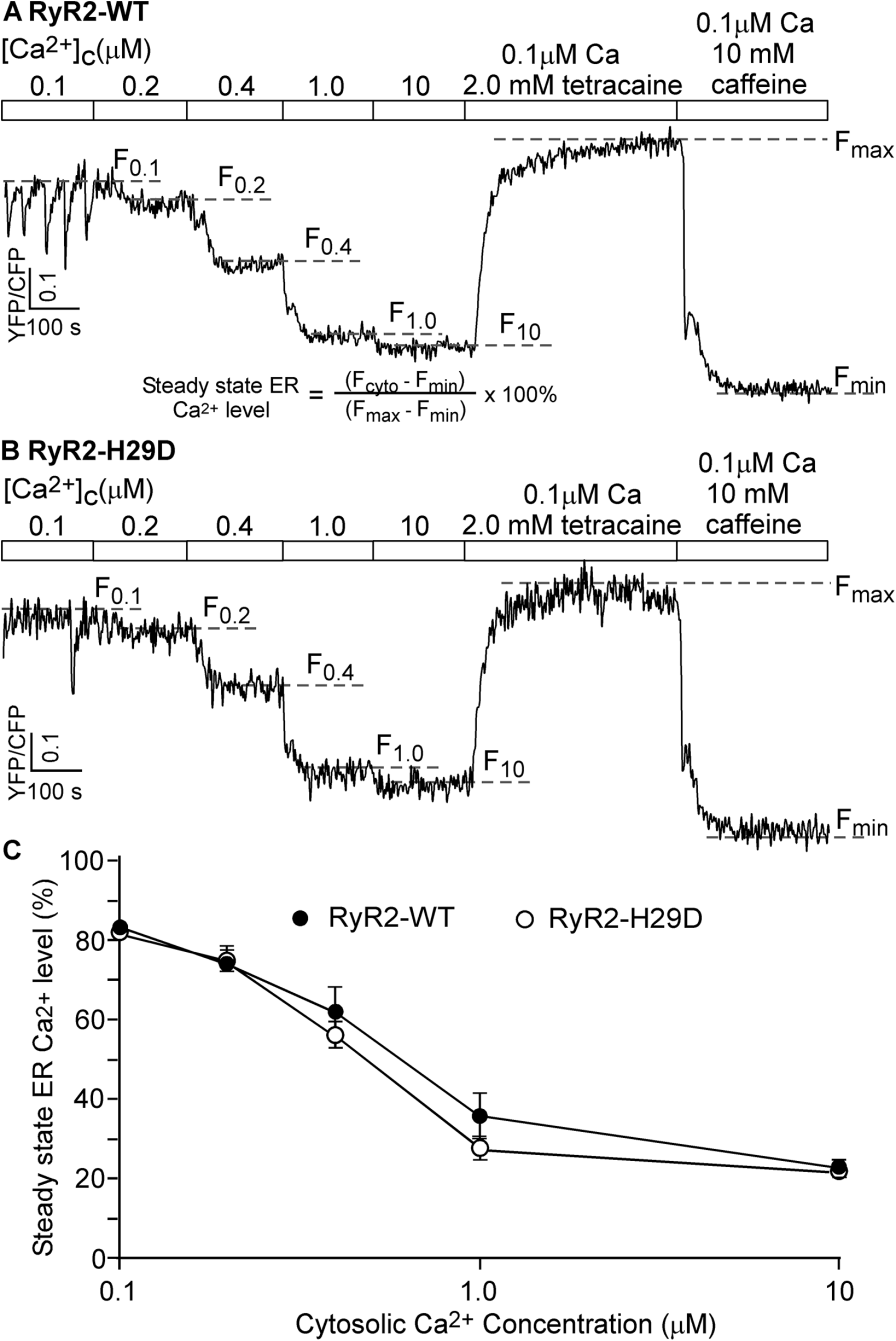
Effect of the H29D mutation on cytosolic Ca^2+^ induced Ca^2+^ release in HEK293 cells. Stable, inducible HEK293 cell lines expressing RyR2 WT (A) or the H29D mutant (B) were transfected with the FRET-based ER luminal Ca^2+^-sensing protein D1ER and their expression were induced using tetracycline. The transfected and induced cells were permeabilized with saponin, washed and perfused with intracellular-like medium plus increasing levels of free cytosolic Ca^2+^ (0.1, 0.2, 0.4, 1.0 and 10 μM) to induce Ca^2+^ release. FRET recordings from representative cells (total 51–93 cells each) are shown. To minimize the influence by CFP/YFP cross-talk, we used relative FRET measurements for calculating the steady state ER Ca^2+^ level (defined in panel A). Dash lines (F_0.1 –_F_10_) indicate the steady state FRET levels after perfusion with each Ca^2+^ concentration (0.1, 0.2, 0.4, 1.0 or 10 μM). The maximum FRET signal F_max_ is defined as the FRET level after tetracaine application. The minimum FRET signal F_min_ is defined as the FRET level after caffeine application. Data shown are mean ± SEM (n = 4–5).

We next assessed the effect of the H29D mutation on the regulation of RyR2 by cytosolic Ca^2+^ in the cellular context. We measured the steady state ER Ca^2+^ level in permeabilized HEK293 cells [34] expressing the RyR2 WT or the H29D mutant at various cytosolic Ca^2+^ concentrations (0.1–10 μM). As shown in Fig 3, incubating permeabilized HEK293 cells expressing RyR2 WT with increasing cytosolic Ca^2+^ concentrations (100nM -10 μM) reduced the steady state ER Ca^2+^ level (Fig 3A and 3C). This reduction in the steady state ER Ca^2+^ level likely reflects cytosolic Ca^2+^ induced fractional Ca^2+^ release from the ER Ca^2+^ store. Consistent with its lack of impact on the Ca^2+^ activation of [^3^H]ryanodine binding and single channels, the H29D mutation did not significantly alter cytosolic Ca^2+^ induced fractional Ca^2+^ release in HEK293 cells (Fig 3C). Thus, these data indicate that the H29D mutation does not alter the cytosolic Ca^2+^ dependent activation of Ca^2+^ release.

### The H29D mutation does not affect the response of RyR2 to caffeine

**Fig 4:**
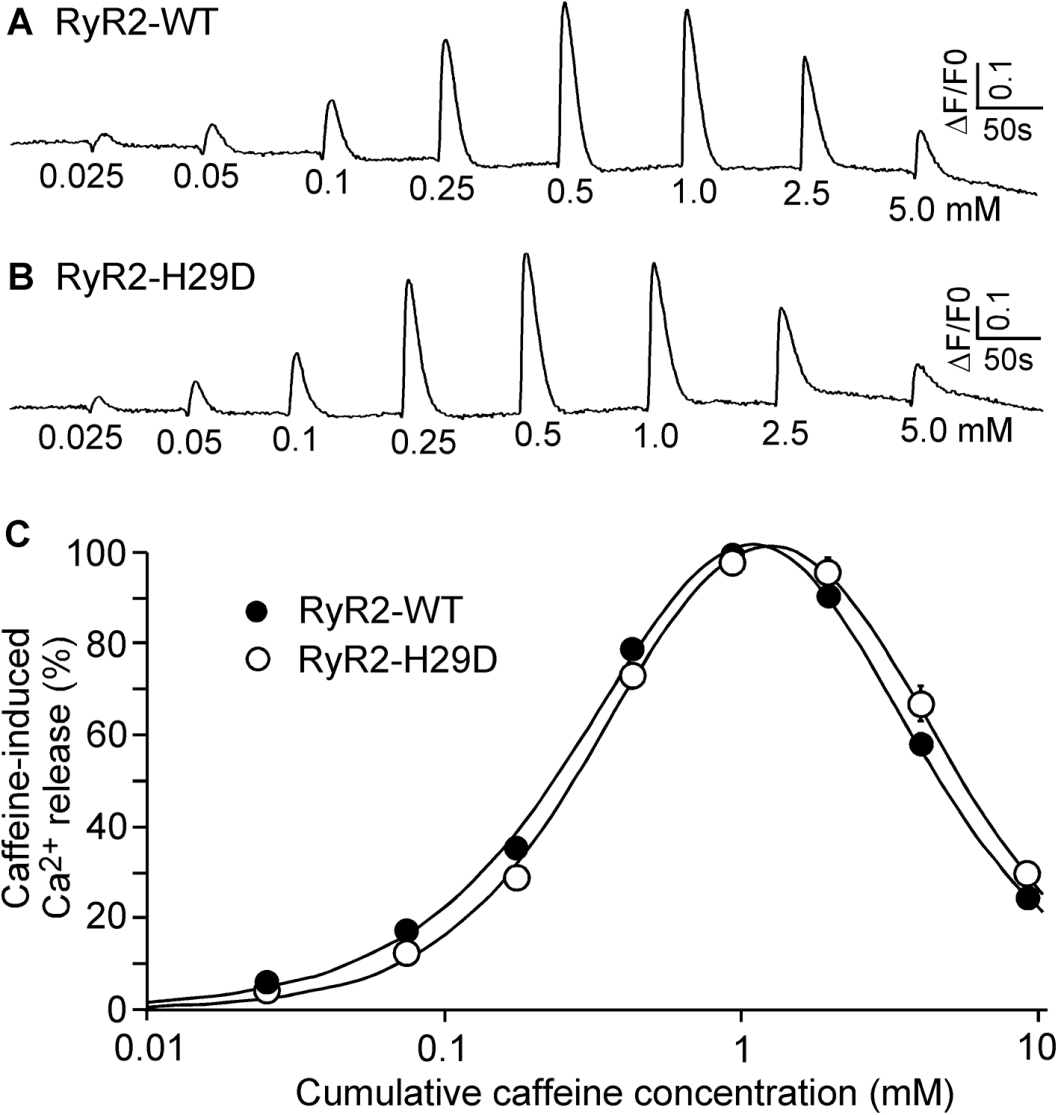
Effect of the H29D mutation on the activation of RyR2 by caffeine. HEK293 cells were transfected with RyR2 WT (A) or the H29D mutant (B). Fluorescence intensity of the Fluo-3 loaded transfected cells before and after additions of increasing concentrations of caffeine (0.025-5mM) was monitored continuously. (C) Ca^2+^ release–Cumulative caffeine concentration relationships in HEK293 cells transfected with RyR2 WT and the H29D mutant. The amplitude of each caffeine peak was normalized to that of the maximum peak for each experiment. Data shown are mean ± SEM (n = 5).

We have previously shown that cytosolic Ca^2+^ and caffeine induce different conformational changes in the structure of RyR2, suggesting different mechanisms of RyR2 activation by these ligands [34]. Although the H29D mutation does not alter the activation of RyR2 by cytosolic Ca^2+^, it is unclear whether it affects the activation of RyR2 by caffeine. To this end, we assessed the responses of HEK293 cells expressing the RyR2 WT and the H29D mutant to caffeine. As shown in Fig 4, applications of incremental concentrations of caffeine (from 0.05 mM to 0.5 mM) induced Ca^2+^ release in HEK293 cells expressing RyR2 WT with increased amplitude. The amplitude of caffeine induced Ca^2+^ release decreased at higher caffeine concentrations (1.0 mM to 5 mM). This decrease likely resulted from the depletion of intracellular Ca^2+^ stores by the prior applications of caffeine (0.025 to 0.5 mM) [36] (Fig 4A). HEK293 cells expressing the H29D mutant displayed caffeine response very similar to that of RyR2 WT expressing cells (Fig 4B and 4C). Thus, the H29D mutation does not affect the activation of RyR2 by caffeine.

### The H29D mutation has no effect on the propensity for spontaneous Ca^2+^ release

**Fig 5:**
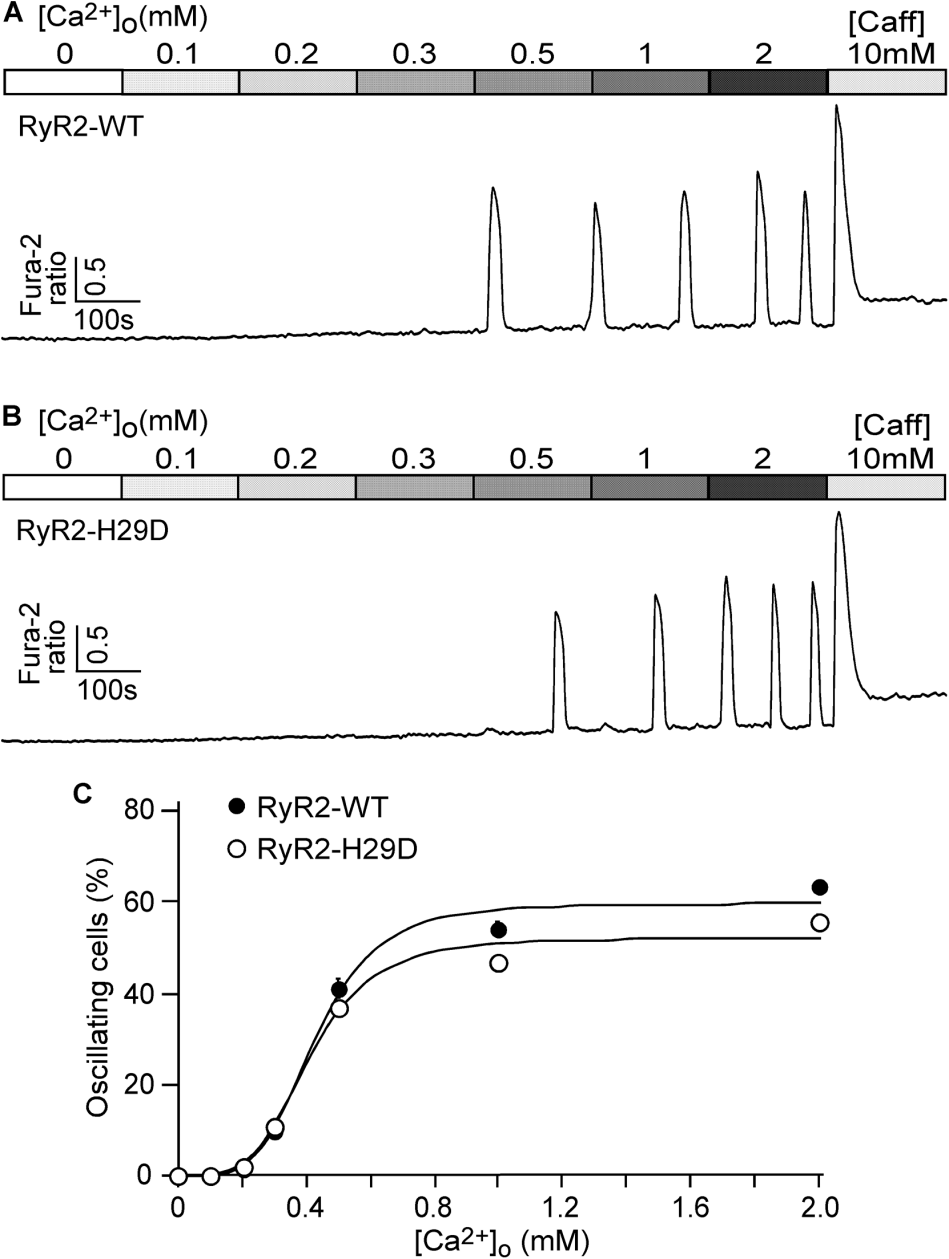
Effect of the H29D mutation on the propensity for SOICR in HEK293 cells. Stable, inducible HEK293 cells expressing RyR2 WT and the H29D mutant were loaded with 5 μM Fura-2 AM in KRH buffer. The cells were then perfused continuously with KRH buffer containing increasing levels of extracellular Ca^2+^ (0–2 mM) to induce SOICR. Fura-2 ratios of representative RyR2 WT (A) and H29D (B) cells were recorded using epifluorescence single cell Ca^2+^ imaging. (C) The percentages of RyR2 WT (total 1087 cells) and H29D (total 1161 cells) cells that display Ca^2+^ oscillations at various extracellular Ca^2+^ concentrations. Data shown are mean ± SEM (n = 9–10).

A common defect of RyR2 mutations associated with CPVT is an enhanced propensity for spontaneous Ca^2+^ release during store Ca^2+^ overload, a process we referred to as store-overloaded induced Ca^2+^ release (SOICR). To determine whether the H29D mutation alters the occurrence of SOICR, HEK293 cells expressing RyR2 WT or the H29D mutant were perfused with elevating extracellular Ca^2+^ concentrations (0–2.0 mM) to evoke spontaneous Ca^2+^ oscillations (SOICR) as described previously [18,26,31]. The resulting SOICR was then studied by single cell Ca^2+^ imaging. As shown in Fig 5, RyR2 WT (Fig 5A) and H29D (Fig 5B) expressing HEK293 cells showed similar percentages of cells that displayed Ca^2+^ oscillations at each extracellular Ca^2+^ concentration (Fig 5C). Thus, these results indicate that the H29D mutation has no effect on the propensity for SOICR.

### The H29D mutation does not alter the SOICR activation or termination threshold

**Fig 6:**
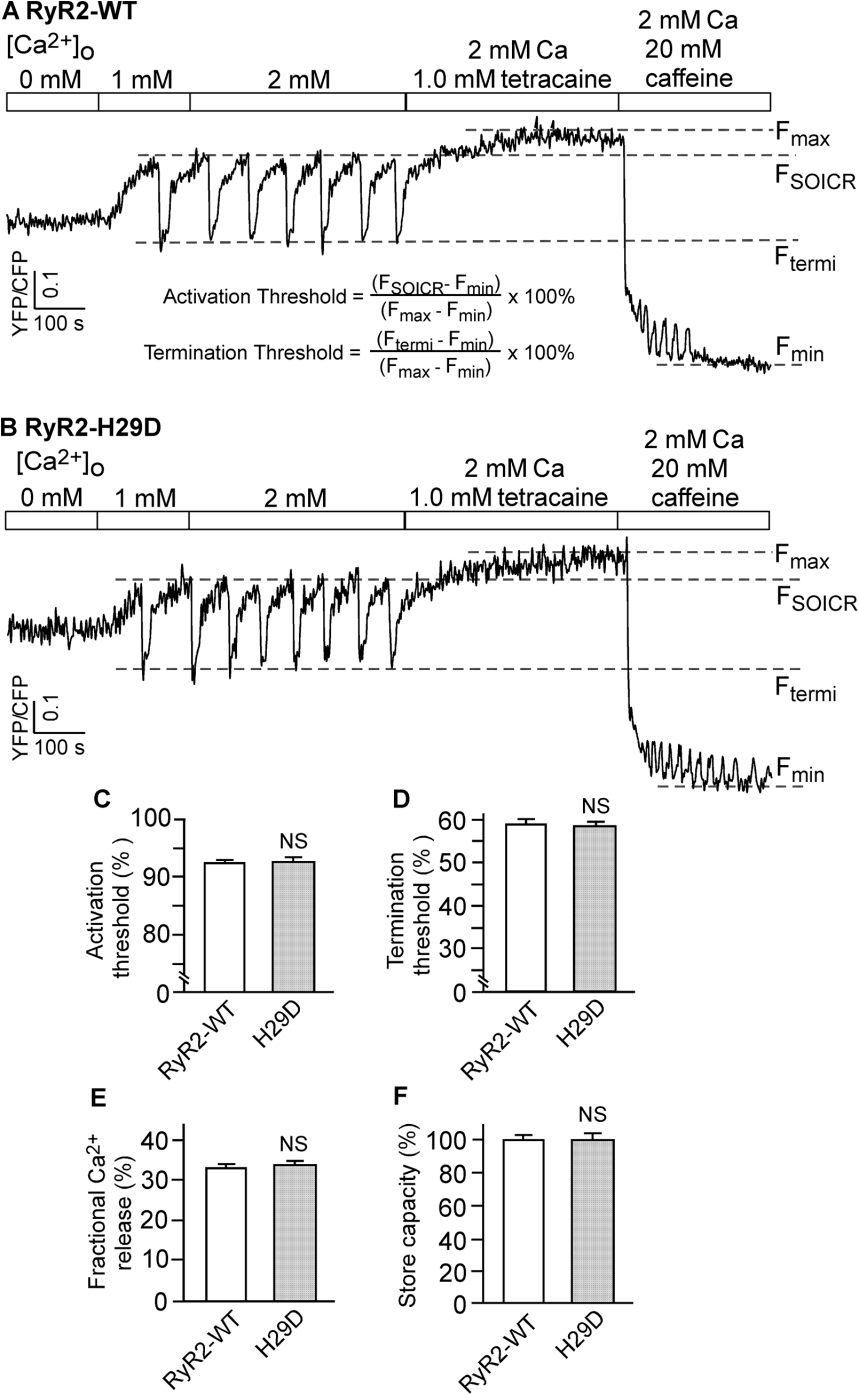
Effect of the H29D mutation on the threshold for SOICR in HEK293 cells. Stable, inducible HEK293 cell lines expressing RyR2 WT or H29D were transfected with the FRET-based ER luminal Ca^2+^ sensing protein D1ER 48 h before single cell FRET imaging. The expression of RyR2 WT and H29D was induced 24 h before imaging. The cells were perfused with KRH buffer containing increasing levels of extracellular Ca^2+^ (0–2 mM) to induce SOICR. This was followed by the addition of 1.0 mM tetracaine to inhibit SOICR, and then 20 mM caffeine to deplete the ER Ca^2+^ stores. FRET recordings from representative RyR2 WT (A) and H29D (B) cells (total 68 cells each) are shown. The activation threshold (C) and termination threshold (D) were determined using the equations shown in panel A. F_SOICR_ indicates the FRET level at which SOICR occurs, while F_termi_ represents the FRET level at which SOICR terminates. The fractional Ca^2+^ release (E) was calculated by subtracting the termination threshold from the activation threshold. The maximum FRET signal F_max_ is defined as the FRET level after tetracaine treatment. The minimum FRET signal F_min_ is defined as the FRET level after caffeine treatment. The store capacity (F) was calculated by subtracting F_min_ from F_max_. Data shown are mean ± SEM (n = 6).

To further assess the effect of the H29D mutation on SOICR, we determined the threshold for SOICR activation and termination in HEK293 cells expressing the RyR2 WT or the H29D mutant. The SOICR activation and termination thresholds were determined by measuring the endoplasmic reticulum (ER) luminal Ca^2+^ levels using D1ER, a fluorescence resonance energy transfer (FRET)-based ER luminal Ca^2+^ sensing protein [32,33]. As shown in Fig 6, HEK293 cells expressing RyR2 WT displayed spontaneous ER Ca^2+^ oscillations upon increasing the extracellular Ca^2+^ concentration from 0 to 2 mM. As reported previously [20,21,33], SOICR occurred when the ER luminal Ca^2+^ content reached a threshold level (F_SOICR_), and terminated when the ER luminal Ca^2+^ content decreased to another threshold level (F_termi_) (Fig 6A). Fig 6B shows SOICR in HEK293 cells expressing the H29D mutant. There was no significant difference in the SOICR activation threshold (H29D: 92.9±0.2% vs. WT: 92.2±0.2%) (Fig 6C) or the termination threshold (H29D: 58.0±0.7% vs. WT: 58.5±1.1%) (Fig 6D). There was also no significant difference in the fractional Ca^2+^ release (H29D: 35.0±0.9% vs. WT: 33.7±0.9%) (Fig 6E) or the store capacity (F_max_−F_min_) between RyR2 WT and the H29D mutant (Fig 6F). Furthermore, SOICR did not occur in control HEK293 cells (without RyR2), and that SOICR was mediated by RyR2, as the IP3R inhibitor, xestospongin C, did not affect SOICR [20]. Therefore, these data indicate that the H29D mutation has no effect on the activation and termination of SOICR.

### The H29D mutation does not affect the thermal stability of the N-terminal region of RyR2

**Fig 7:**
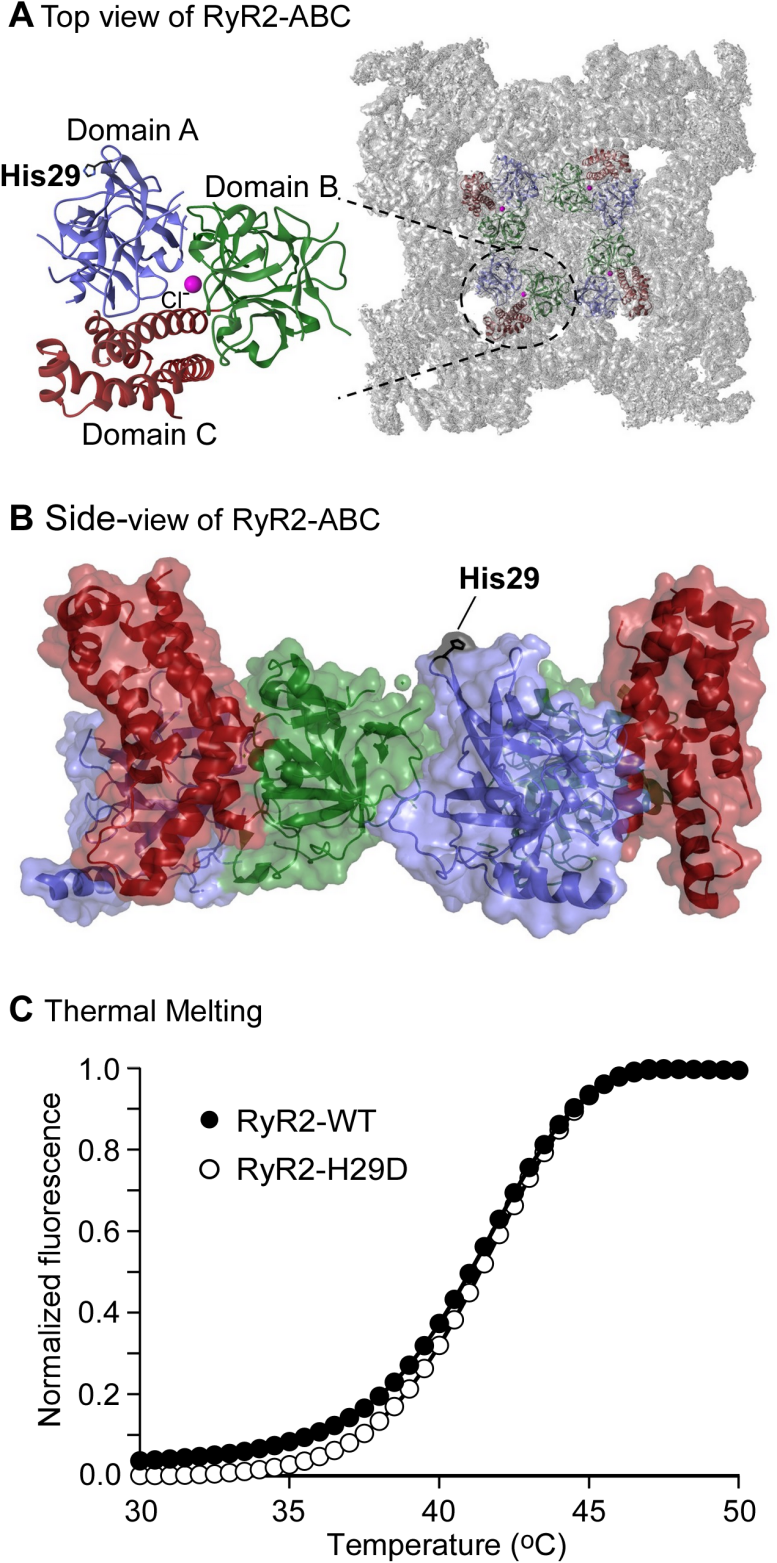
Effect of the H29D mutation on the thermal stability of the N-terminal domains of RyR2. Location of residue H29 in the three-dimensional structure of the N-terminal domains of RyR2 in the top view (A) and side view (B). The central chloride ion is shown as a sphere. Panel A also shows the relative location of the RyR2 N-terminal region within a recent RyR1 cryo-EM map. (C). Representative thermal melting curves of the WT (filled circles) and the H29D mutant (open circles) N-terminal domains of RyR2 (RyR2ABC) (n = 5).

The location of the H29 residue on the surface of the RyR2, away from any interface with other RyR2 domains or known auxiliary proteins (Fig 7A and 7B), supports the lack of functional effects. In previous crystal structures of the RyR2 (1–547) construct, the H29 side chain shows great flexibility, further indicating that it does not form significant interactions with neighboring residues. Disease-causing mutations could also act by causing a general destabilization of the protein fold, which could then indirectly lead to altered domain interactions. Indeed, many disease-causing mutations in RyRs have been found to significantly destabilize the fold, as indicated by a decreased thermal stability [7,12,13,37]. The location at the surface makes this an unlikely scenario for the H29D mutation, but we decided to confirm this by purifying the H29D variant of RyR2 (1–547) and subject it to thermal melt analysis. As shown in Fig 7C, the H29D mutant N-terminal region displayed a temperature dependence of protein unfolding similar to that of the WT N-terminal region. Thus, these data show that the H29D mutation has no significant effect on the stability of the N-terminal domains of the RyR2 channel.

## Discussion

Naturally occurring mutations in the cardiac ryanodine receptor (RyR2) have been linked various cardiac arrhythmias, such as catecholaminergic polymorphic ventricular tachycardia (CPVT), idiopathic ventricular fibrillation, and atrial fibrillation, and structural heart diseases, such as dilated, hypertrophic, and non-compaction cardiomyopathies [2–5]. In order to understand the diverse disease mechanisms associated with RyR2 mutations and to develop mechanism-based strategies for the diagnosis and treatment of RyR2-associated diseases, it is imperative to understand the exact molecular defects of individual RyR2 mutations.

Given their fundamental and clinical importance, over the past decade, a number of structural and functional studies have been carried out to investigate the impact of disease-linked RyR2 mutations. These investigations have provided some important insights into the actions of disease mutations. However, only a small fraction of known RyR2 mutations has been studied. The functional consequences of some of the RyR2 mutations studied are unclear and debatable. For instance, Meli et al. [38] showed that the RyR2-G230C mutation exerts no effect on the Ca^2+^ activation of single RyR2 channels incorporated into planar lipid bilayers under resting conditions. In contrast, we found that single RyR2-G230C channels displayed markedly enhanced sensitivity to Ca^2+^ activation [21]. Furthermore, we showed that the G230C mutation increased the Ca^2+^ dependent activation of [^3^H]ryanodine binding, enhanced the propensity for spontaneous Ca^2+^ release, reduced the threshold for Ca^2+^ release activation and termination, and decreased the thermal stability of the N-terminal region of RyR2 [21]. This G230 residue is part of a conserved Gly-Gly motif that forms a turn at the interface between domains A and B. Replacing G230 with a cysteine (G230C) is likely to affect the geometry of the turn, and thus the relative orientation/packing of domains A and B. In line with this view, our data indicate that the G230C mutation indeed has a dramatic impact on the function and structure of RyR2 [21].

Unlike the G230C mutation, Cheung et al. [23] showed that the H29D mutation significantly enhanced the activation of single RyR2 channels by diastolic cytosolic Ca^2+^. It was proposed that the enhanced activity of the H29D mutant channel at diastolic cytosolic Ca^2+^ would lead to a ‘leaky’ channel under resting conditions, which may explain ventricular tachyarrhythmia occurring in the RyR2-H29D mutant carriers at rest [23]. The H29 residue is located in the loop between beta sheets 1 and 2 in the N-terminal domain A. This loop lies on the surface of the three-dimensional structure of RyR [8,12–16]. Hence, unlike many other disease-linked RyR2 mutations in the N-terminal region, the H29 residue is not located at a domain-domain interface, neither within the N-terminal region nor involving other neighboring domains recently identified in recent cryo-EM studies of RyR [16]. It is also not involved in coordinating the central chloride ion [12], is not located at a known interface with RyR2 auxiliary proteins [17], and is not buried within a domain where it could interfere with folding. On the contrary, the H29 side chain has been found to be highly flexible in multiple crystal structures [12], implying that it doesn’t even interact with side chains of neighboring residues. Given these observations, it is thus puzzling how the H29D mutation could alter the intrinsic properties of the RyR2 channel. To address this important question, we performed a number of functional assays to determine the impact of the H29D mutation. In contrast to those reported by Cheung et al., we found that the H29D mutation does not alter the activation of single RyR2 channels by cytosolic Ca^2+^. Moreover, we showed that the H29D mutation has no effect on the Ca^2+^ dependent activation of [^3^H]ryanodine binding, the propensity for spontaneous Ca^2+^ release, the threshold for Ca^2+^ release activation or termination, or the thermal stability of the N-terminal region of RyR2. Thus, our data on the impact of both the G230C [21] and H29D mutations on single RyR2 channels differ from those of Meli et al. [38] and Cheung et al. [23]. The exact reasons for these controversies are unknown at present. Differences in experimental conditions, such as conditions for transfection, sources/status of RyR2 channels used for single channel studies, and conditions for single channel recordings are likely to be involved. For examples, we used 250 mM K^+^ as the charge carrier for our single channel recordings, whereas Cheung et al. [23] used 53 mM Ca^2+^ as the charge carrier. Furthermore, we characterized the impact of the H29D mutation on the function of the mouse RyR2, whereas Cheung et al. [23] studied the human RyR2. It is possible that the structure of the N-terminal domains in the intact human RyR2 differs slightly from that in the intact mouse RyR2, such that the H29D mutation may affect the function of human RyR2, but not that of mouse RyR2. Clearly, further work will be required to sort out these controversies. Nevertheless, our data obtained from single channel analysis, [^3^H]ryanodine binding and Ca^2+^ release assays, single cell Ca^2+^ imaging, and structural analysis clearly demonstrate that the H29D mutation has little or no impact on the intrinsic function of mouse RyR2 or the structural stability of the N-terminal region.

It is, however, unclear how the H29D mutation that does not alter the intrinsic function of the RyR2 channel causes ventricular tachyarrhythmia at rest. Although the H29D mutation does not affect the isolated RyR2 channel, it may alter the interactions between RyR2 and its regulatory proteins in cardiac cells. The H29 residue is exposed on the surface at the top of the cytosolic assembly of the RyR2 structure. Currently, no auxiliary protein has been shown to interact directly with the RyR N-terminal region, but the possibility remains that such a binding partner exists. This part of the RyR2 structure may interact with proteins associated with the transverse tubular membrane. Thus, it is possible that the RyR2-H29D mutation may alter excitation-contraction coupling or SR Ca^2+^ release by affecting protein-protein interactions that regulate RyR2 function. Further investigations will be needed to determine whether the RyR2-H29D mutation alters RyR2 function and SR Ca^2+^ release in the context of cardiac cells, intact heart or animal models.

Although the H29D mutation has no effect on the cytosolic Ca^2+^ activation of RyR2, whether the H29D mutation has any effect on luminal Ca^2+^ activation of RyR2 has yet to be determined. We have previously shown that a number of CPVT RyR2 mutations enhance the activation of the channel by luminal Ca^2+^ and the propensity for spontaneous Ca^2+^ release upon store Ca^2+^ overload (SOICR) [18,20,26,31]. In the present study, we found that the H29D mutation has no effect on the propensity for SOICR or the threshold for SOICR activation or termination. Given the close link between SOICR and luminal Ca^2+^ activation, the lack of effect of H29D on SOICR suggests that the H29D mutation is unlikely to exert a major impact on luminal Ca^2+^ activation. However, further studies are required to directly assess the impact of H29D on the luminal Ca^2+^ activation of RyR2.

In summary, in the present study, we have characterized the impact of a recently identified RyR2 mutation H29D that is thought to be associated with ventricular arrhythmia at rest using a number of biochemical, electrophysiological, and Ca^2+^ imaging techniques. Our data show that, in contrast to a previous study [23], the RyR2-H29D mutation has no effect on the cytosolic Ca^2+^ activation of RyR2. Our results also demonstrate that the H29D mutation does not alter the propensity for spontaneous Ca^2+^ release, a well-known cause for triggered arrhythmia.

## Abbreviations

RyR2: cardiac ryanodine receptor
CPVT: catecholaminergic polymorphic ventricular tachycardia
SR: sarcoplasmic reticulum
ER: endoplasmic reticulum
PBS: phosphate buffered saline
SOICR: store-overload induced Ca^2+^ release
